# *De novo* genome assembly of *Clonostachys rosea* CMAA1284: a fungal strain with biotechnological potential

**DOI:** 10.1128/mra.00388-26

**Published:** 2026-09-11

**Authors:** Poliana Fernanda Giachetto, Antonio Nhani Junior, Wagner Bettiol, Gabriel Moura Mascarin

**Affiliations:** 1 Embrapa Agricultura Digital 273992 , Campinas, São Paulo, Brazil; 2 Embrapa Meio Ambiente 140635 , Jaguariúna, São Paulo, Brazil; University of California Riverside, Riverside, California, USA

**Keywords:** *Clonostachys rosea*, fungal genomics, *de novo *genome assembly, biological control, plant growth promotion

## Abstract

*Clonostachys rosea* is a fungus with significant applications in biological control, plant growth promotion, and secondary metabolite production essential for agriculture. We report the *de novo* genome assembly of *C. rosea* strain CMAA1284 (also known as LQC 62), which shows high contiguity and excellent gene completeness, supporting its use for functional genomics and biotechnological studies.

## ANNOUNCEMENT

*Clonostachys rosea* is a cosmopolitan filamentous ascomycete widely recognized for its antagonistic activity against plant pathogens, plant growth-promoting ability, and capacity to produce bioactive secondary metabolites and industrial enzymes (1, 2). Genomic resources are essential for functional genomics and strain improvement aimed at enhancing the agricultural and biotechnological potential of this species. Although a reference genome is available (GenBank accession GCA_054828895.1), additional high-quality genome assemblies are needed to better represent intraspecific diversity and facilitate comparative genomic analyses. Here, we report the *de novo* genome assembly of *C. rosea* strain CMAA1284. This native Brazilian strain has been extensively studied by our group, focusing mainly on biological control applications against plant fungal diseases and insect pests, as well as for plant growth promotion (3–5).

Strain CMAA1284 was isolated from rose debris collected in a rose field in Viçosa, Minas Gerais, Brazil (20°45′14″ S, 42°52′55″ W), in 2002 during surveys aimed at identifying biological control agents against *Botrytis cinerea* (6, 7). The strain was cultured in 100-mL potato dextrose broth (Difco, USA) in 250-mL baffled Erlenmeyer flasks at 28°C and 250 rpm for 4 days. Mycelial biomass was harvested, and genomic DNA was extracted using the DNeasy PowerSoil Pro Kit (Qiagen, Hilden, Germany) according to the manufacturer’s instructions. A sequencing library was prepared using the Illumina TruSeq DNA Library Preparation Kit and sequenced on the Illumina NovaSeq platform (2 × 150 bp), generating approximately 64.9 million paired-end reads (9.76 Gb), corresponding to an estimated genome coverage of approximately 168×. Reads were quality filtered and *de novo* assembled using CLC Genomics Workbench v24.0.1 (8). Assembly statistics were generated using CLC Genomics Workbench v24.0.1.

The final assembly comprised 1,266 contigs, totaling 55,190,458 bp, with a mean contig length of 61,119 bp and a largest contig of 1,390,195 bp. The GC content was 50.04%, consistent with previously reported genomes of *Clonostachys* species. Assembly contiguity metrics included an N50 of 243.7 kb (62 contigs). Genome completeness was evaluated using BUSCO v5.8.0 (hypocreales_odb10, 4,494 orthologs). The assembly recovered 96.5% complete BUSCOs, including 95.3% as single-copy and 1.3% as duplicated, with 0.6% fragmented and 2.8% missing BUSCOs, indicating a highly complete genome assembly. Species identity was further confirmed by average nucleotide identity (ANI) analysis using FastANI against publicly available *C. rosea* genomes. The highest ANI value (99.76%) was obtained with *C. rosea* strain NF-06 (GenBank accession GCA_054828895.1), strongly supporting the taxonomic assignment of strain CMAA1284.

The high completeness and contiguity of the CMAA1284 genome assembly provide a reliable foundation for downstream analyses, including functional annotation, secondary metabolite biosynthetic gene cluster prediction, transcriptomic integration, and genome editing strategies. Therefore, this genome assembly represents a valuable genomic resource for both basic and applied research involving *C. rosea*.

## Data Availability

This whole-genome shotgun project has been deposited in GenBank under accession number JBUZWH010000000 (GCA_057376185.1). The associated BioProject and BioSample accession numbers are PRJNA1422825 and SAMN55322612, respectively. Raw sequencing reads are available in the Sequence Read Archive (SRA) under accession number SRR37333998.
